# Method for Determining the Coefficient of Friction Variation Pattern as a Function of Density at Low Temperatures Using the Example of Dry Ice–Steel Contact

**DOI:** 10.3390/ma17102396

**Published:** 2024-05-16

**Authors:** Jan Górecki, Wiktor Łykowski, Jozef Husar, Lucia Knapčíková, Maciej Berdychowski

**Affiliations:** 1 Faculty of Mechanical Engineering, Institute of Machine Design, Poznan University of Technology, 60-965 Poznań, Polandmaciej.berdychowski@put.poznan.pl (M.B.); 2 Department of Industrial Engineering and Informatics, Faculty of Manufacturing Technologies, Technical University of Košice, Bayerova 1, 08001 Prešov, Slovakia

**Keywords:** coefficient of friction, compaction, densification, powders, dry ice, carbon dioxide (CO_2_)

## Abstract

The developments in manufacturing technologies are expected to reduce energy input without compromising product quality. Regarding the material densification process, numerical simulation methods are applied to achieve this goal. In this case, relevant material models are built using functions that describe the variation in mechanical parameters of the material in question due to its deformation. The literature review conducted for this research has revealed a shortage of experimental research methods allowing a determination of the coefficient of friction at low temperatures, approximately 200 K. This article proposes a method for determining the friction coefficient of dry ice sliding against steel. The experimental results were analysed to obtain several functions describing the variation in the coefficient of friction. These functions were then compared using goodness-of-fit indexes. Finally, two functions with similar goodness-of-fit values were chosen. The findings of this research project will complement the already available information and may be used in various research and implementation projects related to the development or improvement of currently used crystallised carbon dioxide conversion processes.

## 1. Introduction

Cold compaction is a process of shaping various pulverised materials, including metallic [1,2], ceramic [3] and pharmaceutical powders [4]. Plastics and biowaste materials are densified at elevated temperatures [5]. To simulate these processes with mathematical models, it is necessary to determine the variation in the mechanical parameters as a function of the changing density of the compacted material. In the case of the Drucker-Prager/Cap [6], Cam-Clay [7] or Mohre-Columb [8] models, these include a variation in the Young modulus, Poisson ratio and coefficient of friction (COF) as a function of density [7]. The required data are, however, not available for many materials, this making laboratory experiments an indispensable part of any research in this area. The relevant research methods reported in the literature allow a determination of the above-mentioned relationships generally at normal or elevated temperatures only [9]. This means that there is a dearth of studies investigating the relevant mechanical parameters at temperatures lower than 210 K.

This is the limit temperature that must not be exceeded by the test rig elements if they are used for testing the mechanical parameters of solid carbon dioxide. This requirement results from the peculiar properties of this material, the temperature of which is −78.5 °C, as indicated by Dzido et al., 2021, and Yan et al., 2020, and which sublimates in atmospheric conditions [10,11].

Solid carbon dioxide is obtained through expansion of liquefied CO_2_ to atmospheric pressure [12]. This adiabatic process removes energy from the material at a high rate, causing a liquid–solid transition. Solid CO_2_ is commonly referred to as dry ice (DI). According to Liu et al., dry ice has a density of 550 kg/m^3^ and is composed of particles of between 20 and 100 μm in size [13]; the smaller they are, the faster dry ice sublimates [14].

In practical applications, it is, therefore, most desirable to reduce the phase transition surface by compacting the powder into pellets or blocks, for example. Compressed DI has found applications in many areas. Zhao et al., 2022, indicated that DI blocks were successfully used for the rapid cooling of food products [15]. The article by Dzido et al., 2023, describes numerical studies on the removal of solid deposits on the surface, using the dry ice blasting method, in which external coatings were removed by hitting the surface with DI pellets, which rapidly decompressed while penetrating into gaps between the coating and the substrate [16].

**Figure 1:**
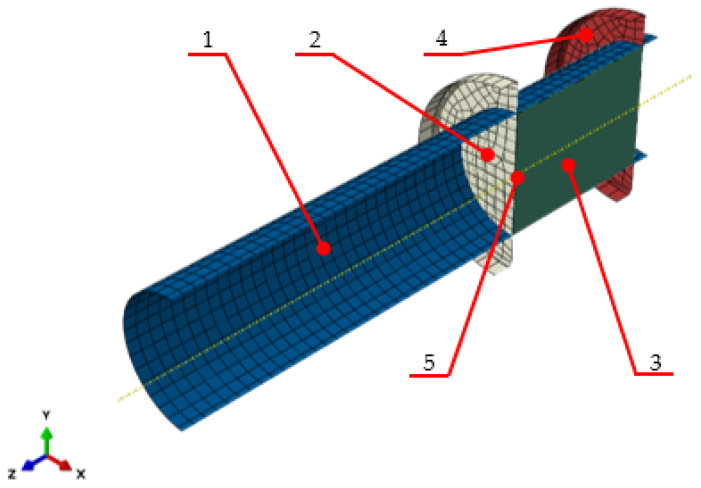
Model used to simulate dry ice compaction in a closed chamber: 1. barrel, 2. ram, 3. extruded dry ice, 4. dead-end disc, and 5. force measurement point [17].

Mathematical models describing the compaction of dry ice have been published in previous studies by the authors of [17,18]. However, during simulation studies, simplification was carried out, assuming that the friction coefficient (*μ_W_*) during DI contact (Figure 1; label 1) with the die cavity working surfaces (Figure 1; label 2) of μ = 0.05 did not change with the changing density of the material being compacted. In reference papers, the coefficient factor was formulated according to Coulomb’s model, where the friction coefficient was described as the ratio of the force normal to the surface and the tangential force. As a result of this physical relationship, the friction coefficient has no assigned unit.

**Figure 2:**
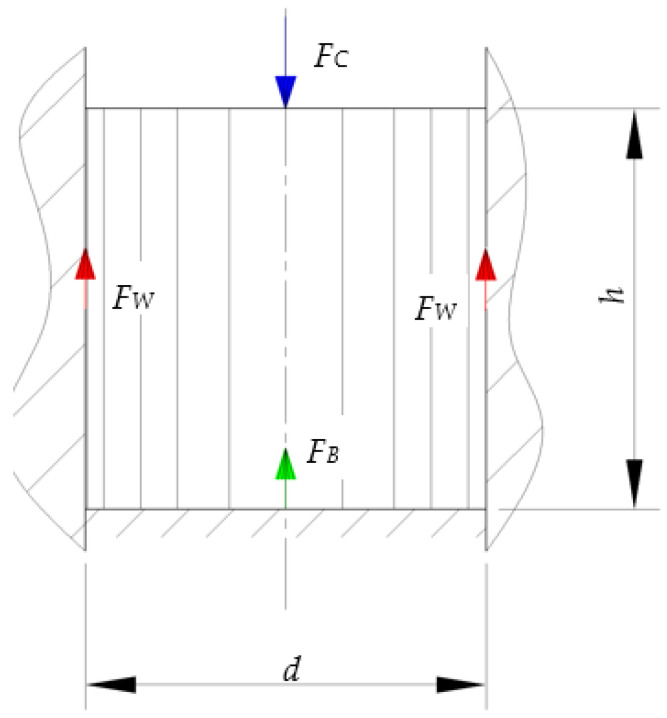
Distribution of forces during extrusion of a pulverised material in a closed chamber. *d*—diameter of the compaction chamber; *h*—specimen height.

In the study on plastic compaction by T. Vu et al., 2020, a significant effect of the degree of compaction on the value of the coefficient of friction was reported [19]. This finding was confirmed in the studies by Briscoe et al., 1991, and Saloda et al., 2013 [20,21]. Yusof et al., 2010, presented the following equation describing the relationship between the COF value against the die cavity surfaces, *F_W_*, and two process parameters: the compaction force, *F_C_*, and force transmitted by the extruded material to the die cavity end, *F_B_* (Figure 2) [22].

(1) $$F_{W}=F_{C}-F_{B}.$$

Briscoe et al., 1991 [23], pointed out that as regards the compaction of ceramic powders, the coefficient of friction against the die cavity walls, *μ_W_*, can be determined using the following equation:

(2) $$ln\left(\frac{\sigma_{B}}{\sigma_{C}}\right)=-4\mu_{w}K_{W}F_{D}\left(\frac{h}{d}\right),$$

where the logarithmic value of the ratio of compression stress on the bottom of the chamber, *σ_B_*, from the compacting *σ_C_* is equal to four times the value of the product of the coefficient of friction, *μ_W_*, and the following apply:

*K_W_*—radial to axial stress ratio [23];

*F_D_*—distribution factor dependent on the normal-to-transmitted-stress ratio, calculated when transmitted stress reached the asymptotic value, which actually occurred in many systems, and the ratio between the specimen height, *h*, and its cross-sectional diameter, *d* [22].

**Figure 3:**
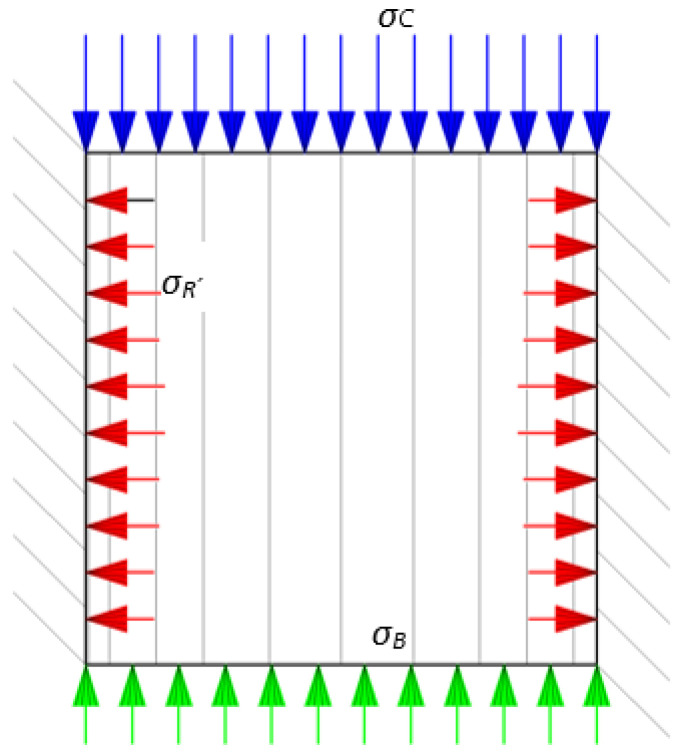
Example of stress distribution when compacting pulverised material in a closed chamber; *σ_R_*—radial stress of a compacted material in a cylindrical chamber.

The distribution of the respective stresses is illustrated in Figure 3 below.

The above equation gives the value of the natural logarithm of the stress ratio as the product of constants (parameters) used. As a result, it does not take into account the change in the properties of individual parameters as a function of material density. Therefore, the *μ_W_* value obtained with the Briscoe model does not allow the numerical models developed in the literature to be expanded with the variability, *μ_W_*, as a function of the density of the material being compacted.

Brewin et al., 2008, pointed out the possibility that when compressing cylindrical specimens in closed chambers, where the ratio of height to diameter is between 1 and 1.5, it can be assumed that the radial stress value in each direction of the Cartesian system is similar [24], which can be represented by the following equation:

(3) $$\varepsilon_{W}=\frac{1}{E}\left(\sigma_{R}-\nu \left(\sigma_{C}+\sigma_{R}\right)\right)=0,$$

(4) $$\nu =\frac{\sigma_{R}}{\sigma_{C}+\sigma_{R}},$$

where *ε_W_*—absolute radial strain; *E*—young modulus; ν—Poisson ratio.

Knowing the geometric parameters of specimens *h* and *d*, described in Figure 1, and the above relationship in σ_C_, σ_B_ and σ_R_, based on the Coulomb friction model, Equation (1) can be converted into the following form:

(5) $$Fw=\mu_{w}h\frac{4F_{C}}{d}\cdot \frac{1-\nu }{\nu }.$$

Although this equation represents a simplification of the phenomenon of friction during the compaction of pulverised materials, it has been applied with success in many other cases [25].

In order to account for the change in the Poisson’s ratio, *ν*, of the material being compacted, the equation (Equation (5)) was modified by replacing the constant value of *ν*, with the mathematical relationship *ν*(*ρ*), describing a change in its value as a function of density, *ρ*.

(6) $$Fw\left(\rho \right)=4\mu_{w}F_{C}\frac{h}{d}\cdot \frac{1-\nu \left(\rho \right)}{\nu \left(\rho \right)}.$$

The change in *ν* as function of *ρ* was described by Biszczanik et al., 2022 [26]. In the following chapter dealing with the analysis of experimental results, we compare the goodness of fit of various mathematical functions used for the regression of the studied variations. On this basis, we proposed representing the variation in *ν*(*ρ*) with the following function:

(7) $$\nu \left(\rho \right)=-7.12\cdot 10^{-2}\;\frac{0.59\;\rho^{8.22}}{1.220^{8.22}+\rho^{8.22}}\;.$$

The system of Equations (1) and (6) gives the value of the coefficient of friction, *μ_W_*, based on the empirical values of *F_C_*, *F_B_* and *ρ*.

This article complements the existing research gap in the measurement of the dry ice friction coefficient and its change as a function of density. The research methodology presented in the article together with the description of the results allow us to fill the identified research gap. The proposed function can be used in numerical simulations carried out using both discrete and finite element methods.

## 2. Materials and Methods

### 2.1. Materials

**Figure 4:**
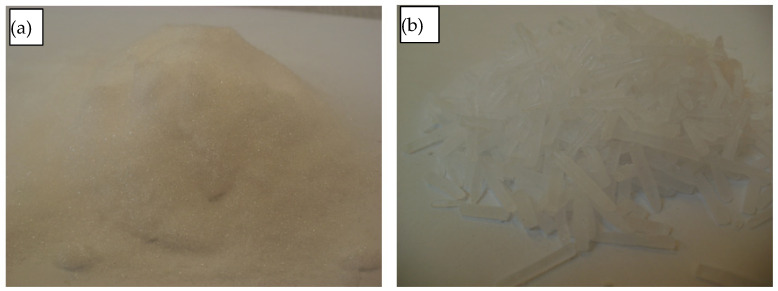
Dry ice: (**a**) pulverised dry ice; (**b**) dry ice extruded into pellets.

The material used in this study was pulverised crystalline carbon dioxide (CCD). Pulverised CCD is obtained by expansion to the atmospheric pressure of liquid CO_2_, which is stored in special tanks at a pressure of approx. 18 bar [27,28]. Liu et al., 2012, indicated that the maximum particle size of this material is 100 µm [7]. It is a loose material, with a bulk density of 550 kg/m^3^; see Figure 4. Bisczczanik et al., 2021, in their study on Young’s modulus variation as a function of density, showed that the limit of material density is about 1650 kg/m^3^ [29,30]. Considering the low cohesiveness of the material, the researchers decided not to measure this parameter for densities below 1000 kg/m^3^.

Taking into account the properties of pulverised CCD, specifically its high sublimation rate [31], the material was stored in special polystyrene foam containers with 40 mm thick walls.

**Figure 5:**
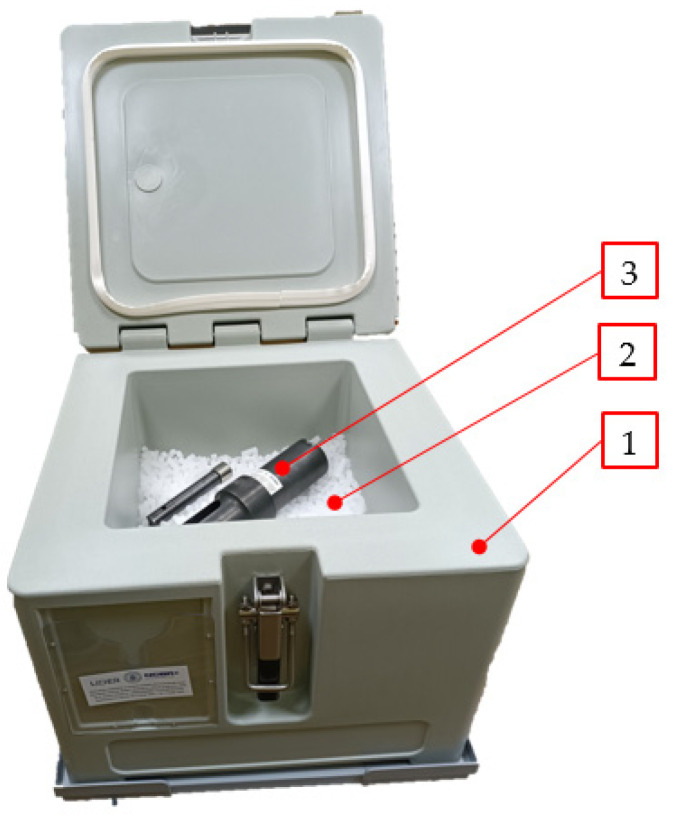
Insulated container used to store pulverised dry ice and cool down the test rig elements used in this research. 1—insulated container; 2—dry ice; 3—test rig [31].

In addition, the test rig parts, as described in a later part of this article, were cooled to the temperature approximating the CCD temperature. For this purpose, they were placed in a DRICY 30L dry ice storage container manufactured by Melform of Monasterolo di Savigliano, Italy (Figure 5). The desired cooling effect was achieved by filling the container with dry ice pellets (Figure 4b).

### 2.2. Method

The method described by Brewin et al., 2008 [24], was used to determine the CCD coefficient of friction values depending on the density of the material in question. In this method the friction force, *F_W_*, is determined through the simultaneous measurement of the force applied on the ram, *F_C_*, and the force acting on the die cavity bottom, *F_B_*. Now, taking Equation (1) we can calculate the *F_W_* value by deducting the latter from the former value.

**Figure 6:**
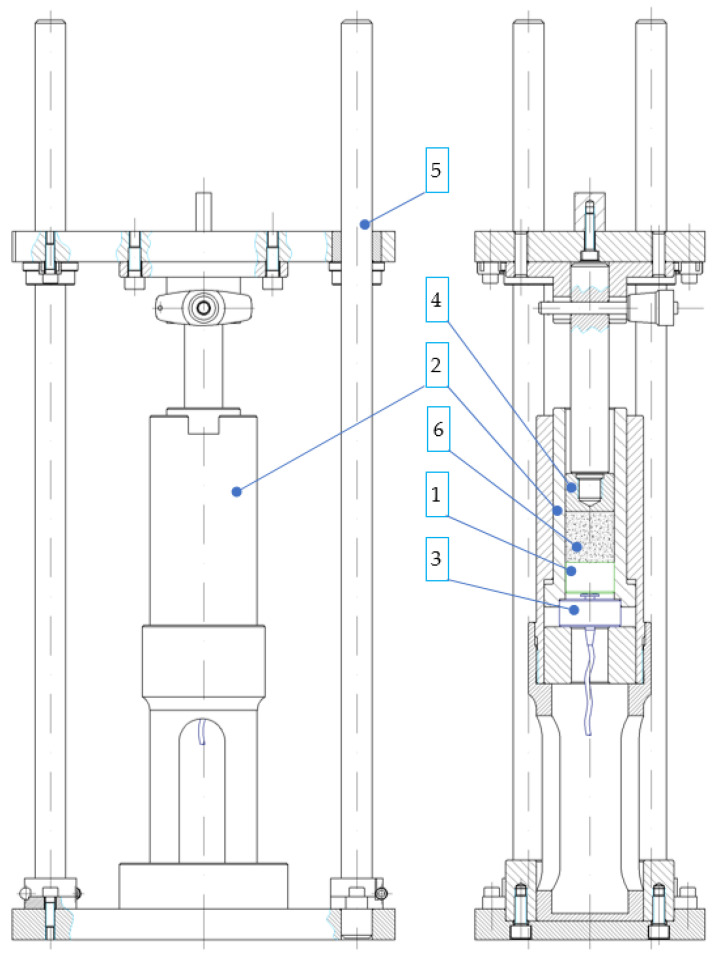
Test rig for measuring the variation in the static friction coefficient as a function of dry ice density. 1. Moveable bottom of the die cavity; 2. die cavity; 3. strain gauge fitted under the die cavity bottom; 4. ram; 5. guide system; 6. crystalline carbon dioxide.

In order to measure these values, the test rig described by Biszczanik et al., 2021 (Figure 6) [29], was modified by replacing the die cavity bottom with a movable plastic piston (Figure 6, label 1). The piston could move reciprocally inside the die cavity (Figure 6, label 2) up to the C9C 10 kN load cell manufactured by HBM, Villingen-Schwenningen, Germany (Figure 6, label 3), fitted at the bottom.

The test rig was mounted in the jaws of an Isight 50 kN universal testing machine manufactured by MTS, Boston, MA, USA, which has a special guide system (Figure 6, label 4) to ensure the coaxial movement of the ram in relation to the die cavity axis.

Before the tests, the machine complete with the C9C strain gauge and ram was kept for 60 min in a dry-ice-filled container. The test rig was re-cooled for 10 min after a maximum of three tests conducted in a series to maintain the desired low temperature of the rig components, in order to reduce the influence of CCD sublimation on the measurement results. The tests were carried out in controlled conditions at an ambient temperature of 18 ± 2 °C. The temperature of inside surfaces was also checked, and it was found to increase by ca. 10 K.

During the tests, the C9C and MTS output signals were processed on a Spider 8 amplifier manufactured by HBM, Villingen-Schwenningen, Germany. Catman Easy program, version 3.5, manufactured by HBM, was used for data acquisition at a 100 Hz sampling frequency. The simultaneous logging of both forces and the load point displacements allowed us to determine the variation in the difference between these two forces as a function of displacement.

Before the tests, the cooled die cavity was filled with crystalline carbon dioxide portions (Figure 6, label 6), the respective weights of which are given in Table 1 below. Next, the rig was fitted in the bottom socket of the ram guide system. With all the elements in place, the test was started using the TestWork 4.0 program. In the first phase, the ram was moved down at a speed of 5 mm/s. until the compaction force increased up to at least 100 N. Next, the ram continued traveling at a load point displacement rate of 0.05 mm/s. The ram stopped when the distance between its front face and the bottom piston reached or exceeded 20 mm, equal to the temporary height of the compressed material. Finally, the ram withdrew to its home position, thus ending the process of compaction.

Now, with the displacement values, *h* (mm), and knowing the die cavity diameter of *D_S_* = 20 mm, we can calculate the volume at the end of test. The sample was removed from the tester, and its weight, *m*_2_ (g), was measured to 0.001 accuracy using the Axis ACN220 analytical balance manufactured by Axis, Gdańsk, Poland. These values were substituted into the following equation to calculate the densities of the respective specimens:

(8) $$\rho_{s}=\frac{4\cdot m_{2}}{h\cdot \pi \cdot {D_{S}}^{2}}\;\left[\frac{g}{mm^{3}}\right]$$

### 2.3. Test Rig Calibration

The C9C 10 kN output signal was read within the temperature range specified by the manufacturer, i.e., −65 °C to −78 °C. An additional test was done to verify the readings and derive a signal correction function if required.

During the tests, the rig shown in Figure 6 was cooled down as described earlier in this article. Next the extrusion barrel assembly, complete with C9C sensor was fitted in the ram guide system. The load point of the tester was then moved to obtain 0.5 mm distance between the ram front face and the C9C tip. Finally, the readings from the respective sensors were reset to zero. The test procedure, using the Test Work program could now be executed. In this process the ram moves vertically at a constant speed of 0.1 mm/s. When the force value reached 0.5 kN, the ram travel speed was reduced to 0.01 mm/s. The ram was stopped when the force reading obtained with the strain transducer fitted on the universal test machine reached 9 kN.

Throughout the test, the output signals from both load cells were recorded using an Spider 8 amplifier (manufactured by HBM, Villingen-Schwenningen, Germany_and stored in the dedicated CatMan Easy (version 3.5) software program. The above-described test procedure was repeated 20 times.

The recorded values showed a difference between the two load cell readings. Considering the surrounding environment conditions, the reading of the MTS Insight 50 kN load cell was taken as the reference value. This value was then used to determine the correction factor, calculated as the average ratio between the maximum force readings of HBM C9C 10 kN and MTS Insight 50 kN load cells.

(9) $$k=\frac{1}{20}\sum_{n=1}^{20}\frac{F_{n}^{C9C}}{F_{n}^{Insight}}=1.049$$

## 3. Results and Discussion

**Figure 7:**
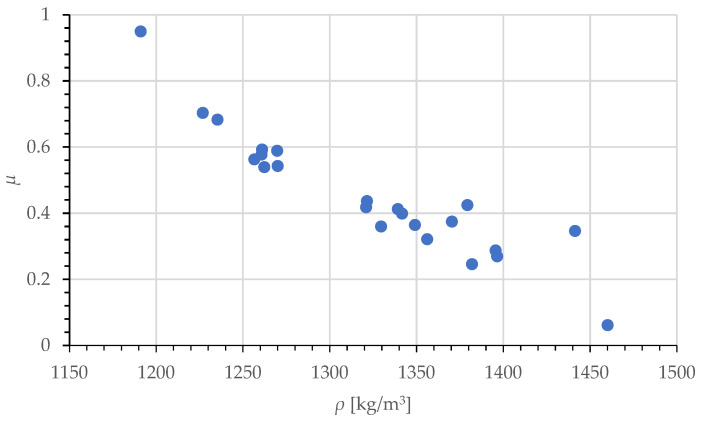
Distribution of COF values as a function of *ρ*, calculated based on the obtained experimental results.

The outcome of this research is presented in the following chart (Figure 7). Despite the repeatable material feed maintained for the four populations, it was not possible to relate the feed weights to their corresponding populations due to large scatter of the end product densities. This being so, for the purposes of further analysis, we decided to combine the populations ascribed to the feed weight, m_0_, into one data set. The regression value was ca. 0.928, calculated using the Pearson correlation coefficient. However, the literature review [32,33] does not support the regression in the experimental results with the use of linear functions. Therefore, further analysis was limited to non-linear functions with a goodness of fit index higher than that of the linear function, i.e., R^2^ = 0.86, and a standard error below 0.07.

The regression values were calculated using the CurveExpert Professional software, version 2.7.3, program by D. Hyams, version 2.7.3. The following non-linear functions were used in the analysis: exponential (10), geometric (11), sinusoidal (12), and natural logarithmic (13). The equations relevant to these functions are described below. These functions allow regression, taking into account the assumption related to the physical interpretation of the friction coefficient, the value of which should fall in the range from 0 to 1. In all of the indicated equations, the variable *ρ* was determined and expressed in kg/m^3^.

(10) $$\mu =a{\cdot e}^{b\rho },$$

(11) $$\mu =a{\cdot \rho }^{b\rho },$$

(12) μ=a+b·cos⁡(c·ρ+d),

(13) $$\;\mu =a+b\cdot ln\left(\rho \right).$$

The calculated parameters of the respective functions are given in Table 2 below.

**Figure 8:**
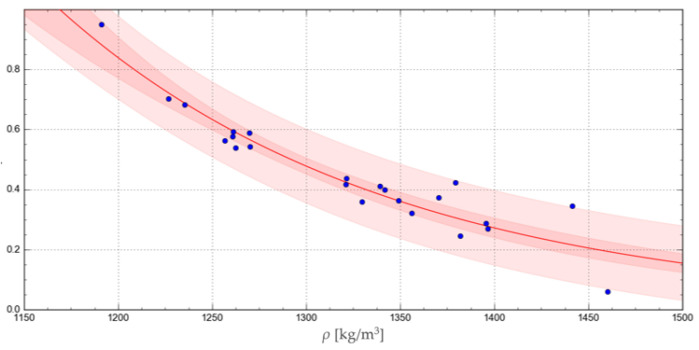
Exponential curve representing the change in the *μ* value as a function of *ρ*, showing the relevant confidence and prediction band.

**Figure 9:**
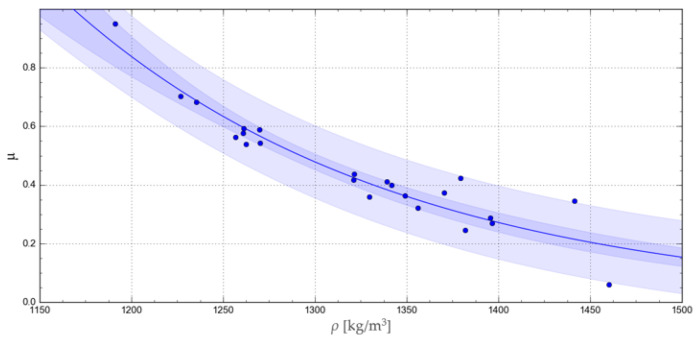
Geometric curve representing the change in the *μ* value as a function of *ρ*, showing the relevant confidence and prediction band.

**Figure 10:**
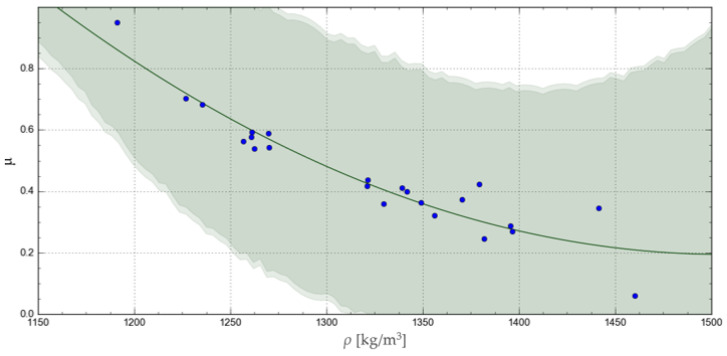
Sine curve representing the change in the *μ* value as a function of *ρ*, showing the relevant confidence and prediction band.

**Figure 11:**
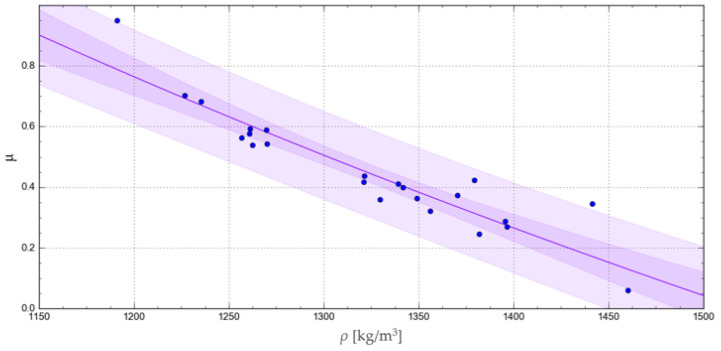
Natural logarithmic curve representing the change in the *μ* value as a function of *ρ*, showing the relevant confidence and prediction band.

The accuracy of representation of the experimental results by the relevant functions was verified using the R-squared coefficient of determination, Akaike information criterion, corrected for a small samples (AICc) [34], and the confidence and prediction band, as satisfactorily used by Shah M. et al., 2017, in catchment runoff mathematical modelling [35]. The curves obtained with the respective functions are represented in Figure 8, Figure 9, Figure 10 and Figure 11, together with the corresponding confidence and prediction bands, and the goodness of fit indexes are given in Table 3.

Among all these functions, the highest R-squared values were obtained for the exponential and geometric functions. In turn, a comparison of the confidence and prediction bands showed no significant differences between the applied regression functions. Finally, the lowest AICc test value was obtained for the exponential function, indicating the best goodness of fit with the experimental results.

Middelgof et al., 2020, and Ku et al., 2023, demonstrated the exponential nature of the relationship between mechanical parameters and the density of porous materials [36,37]. The results of our analysis and the above-mentioned literature allow us to conclude that exponential and geometric functions yield similar results, appropriate for the variation pattern of this parameter.

## 4. Conclusions

The proposed method was found to be an effective tool for the determination of the static coefficient of friction as a function of the changing density of dry ice sliding on steel. Similarly to the findings of published studies on other pulverised materials, mechanical parameters such as the Poisson factor or Young modulus value evolve as a result of compaction, up to the limit density of the material in question.

Chapter 3 presents four mathematical models that were used in this research to represent the variation in *μ* as a function of *ρ*. Two of them are characterised by a very high goodness of fit index value and a low AICc value at the same time. For the maximum density of *ρ* = 1625 kg/m^3^, the values of ca. 0.07 and ca. 0.08 were obtained with the exponential (Equation (10)) and geometrical (Equation (11)) functions, respectively. In addition, for *ρ* in the range from 1200 to 1625 kg/m^3^, the *μ* values obtained with these two functions did not differ by more than 0.02, and this difference tended to decrease with increasing density, *ρ*. Thus, both models were found to feature adequate goodness of fit with the experimental data and low AICc values.

This finding is particularly significant because, on this basis, we can now represent the variation in *μ* depending on the density of extruded dry ice. This allows a prediction of the behaviour of this material at different degrees of compaction. The interest in the practical application of this material in various industries has been growing recently, as previously mentioned in Chapter 1. Furthermore, the findings of this research may turn to be useful in future studies of geological and construction aspects related to the planned exploitation of the planet Mars [38,39].

The authors also intend to apply the obtained results in studies on modelling the failure of compacted dry ice, to obtain a more reliable representation of DI extrusion through multiple cavity dies. For this purpose, at this stage, we are considering using the modified Doraivel model that was used by Xu et al. in 2022 to the model cold compaction in the PEKK (Poly-Ether-Ketone-Ketone) powder cold compaction process [40].

## Figures and Tables

**Table 1:** Feed weights before tests. m_0_^I^—specimen mass form group 10 g; m_0_^II^—specimen mass form group 12 g; m_0_^III^—specimen mass form gruop 14 g; m_0_^IV^—specimen mass form group 16 g.

No.	m_0_^I^ [g]	m_0_^II^ [g]	m_0_^III^ [g]	m_0_^IV^ [g]
1	10.1	12.0	14.2	16.1
2	10.0	12.1	14.0	16.2
3	9.9	12.0	14.1	16.2
4	10.0	12.1	14.1	16.0
5	10.1	11.9	14.3	16.0
6	10.0	12.0	14.2	16.1
7	10.0	12.0	14.2	16.1
8	9.9	12.0	14.1	16.1
9	10.1	11.8	14.2	16.0
10	10.1	11.9	13.9	16.0

**Table 2:** Parameters of the functions used for determination of regression of *μ* variation as a function of *ρ*.

	Exponential	Geometric	Sinusoidal	Natural Logarithmic
*a*	7.16·10 ^2^	2.93·10 ^2^	6.79	2.36
*b*	−5.62·10 ^−3^	−6.89·10 ^−4^	6.77	−3.23
*c*	-	-	4.44·10 ^−4^	-
*d*	-	-	8.76	-

**Table 3:** Values of the criteria used to verify the goodness of fit between the regression functions and experimental results.

	Exponential	Geometric	Sinusoidal	Natural Logarithmic
*R* ^2^	0.95	0.95	0.94	0.93
*R*	0.91	0.91	0.90	0.87
*AICc*	−131.04	−130.93	−123.77	−123.43
*Standard Error*	0.057	0.059	0.064	0.068

## Data Availability

Data are contained within the article.

## References

[1] Thirupathi N., Kore S. (2023). Experimental and numerical studies on electromagnetic axial powder compaction of Al 6061 powder. Powder Technol..

[2] Bai Y., Li L., Fu L., Wang Q. (2021). A review on the high velocity compaction mechanism of powder metallurgy. Sci. Prog..

[3] Kumar N., Baharti A., Dixit M. (2021). Powder Compaction Dies and Compressivity of various Materials. Theory Technol. Form. Process.

[4] Cabiscol R., Shi H., Wünsch I., Magnanimo V., Finke J.H., Luding S., Kwade A. (2020). effect of particle size on powder compaction and tablet strength using limestone. Adv. Powder Technol..

[5] Wilczyński D., Talaśka K., Wojtkowiak D., Górecki J., Wales K. (2024). Energy consumption of the biomass cutting process anticipating the biofuel production. Biosyst. Eng..

[6] Buljak V., Baivier-Romero S., Kallel A. (2021). Calibration of Drucker–Prager Cap Constitutional Model for Ceramic Powder Compaction through Inverse Analysis. Materials.

[7] Diarra H., Mazel V., Busignies V., Tchoreloff P. (2017). Comparative study between Drucker-Prager/Cap and modified Cam-Clay models for the numerical simulation of die compaction of pharmaceutical powders. Powder Technol..

[8] Cricrì G., Perrella M. (2016). Modelling the mechanical behavior of metal powder during die compaction process. Fract. Struct. Integr..

[9] Wang Y., Li C., Jia T., Pan S. (2024). a parameters identification method and experimental analysis of contact friction interface in thermal environment. Mech. Based Des. Struct. Mach..

[10] Dzido A., Krawczyk P., Badyda K., Chondrokostas P. (2021). Operational parameters impact on the performance of dry-ice blasting nozzle. Energy.

[11] Yan L., Huang J., Guo Z., Dong X., Wang Z., Wang Y. (2020). Solid-State Proton Battery operated at Ultralow Temperature. ACS Energy Lett..

[12] Gorecki J., Lindner T., Walesa K. (2023). Evaluation of Density Fields of Numerical Analysis Output of Solid Carbon dioxide Extrusion process. Adv. Sci. Technol. Res. J..

[13] Liu Y., Calvert G., Hare C., Ghadiri M., Matsusaka S. (2012). Size measurement of dry ice particles produced form liquid carbon dioxide. J. Aerosol Sci..

[14] Biszczanik A., Wojtkowiak D., Wałęsa K. Influence of the geometric parameters of convergent extrusion channel of die on the maximum value of compaction stress of dry ice and on the quality of the obtained pellets. Proceedings of the 27th International Polish-Slovak Conference on Machine Modelling and Simulations 2022.

[15] Zhao Y., Ning J., Sun Z. (2022). Establishment and experimental verification of temperature prediction model for quick-frozen strawberry jetted with dry ice. Food Process Eng..

[16] Dzido A., Krawczyk P. (2023). Abrasive Technologies with Dry Ice as a Blasting Medium—Review. Energies.

[17] Berdychowski M., Górecki J., Wałęsa K. (2022). Numerical Simulation of Dry Ice Compaction process: Comparison of the Mohr–Coulomb Model with the Experimental Results. Materials.

[18] Walesa K., Górecki J., Berdychowski M., Biszczanik A., Wojtkowiak D. (2022). Modelling of the process of Extrusion of Dry Ice through a Single-Hole Die using the smoothed Particle Hydrodynamics (SPH) Method. Materials.

[19] Vu T., Nezamabadi S., Mora S. (2020). Compaction of elastic granular materials: Inter-particles friction effects and plastic events. Soft Matter.

[20] Briscoe B.J., Rough S.L. (1998). The effects of wall friction in powder compaction. Colloids Surf. A Physicochem. Eng. Asp..

[21] Saloda M.A. (2013). To Study the influence of Frictional conditions and Die Land Length on Component Error and Die Deflecion in Cold Extrusion by Finite Element Analysis. J. Metall. Eng..

[22] Yusof Y., Ng S., Chin N., Talib R. (2010). Compaction pressure, wall friction and surface roughness upon compaction strength of Andrographis paniculata tablets. Tribol. Int..

[23] Briscoe B.J., Evans P.D. (1991). Wall friction in the compaction of agglomerated ceramic powders. Powder Technol..

[24] Brewin P., Coube O., Doremus P., Tweed J. (2008). Modeling of Powder Die Compaction.

[25] Wilczyński D., Berdychowski M., Talaśka K., Wojtkowiak D. (2021). Experimental and numerical analysis of the effect of compaction conditions on briquette properties. Fuel.

[26] Biszczanik A., Górecki J., Kukla M., Walesa K., Wojtkowiak D. (2022). Experimental investigation on the effect of Dry Ice Compression on the Poisson Ratio. Materials.

[27] Jassim A., Khalaf H. Dry Ice Cleaning based Sustainable Cleaning Technology for Oil and Gas Storage Tanks. Proceedings of the 1st International Multi-Disciplinary Conference Theme: Sustainable Development and Smart Planning.

[28] Mikołajczak A., Krawczyk P., Kurkus-Gruszecka M., Badyda K. (2019). Analysis of the Liquid Natural Gas Energy Storage based on the mathematical model. Energy Process.

[29] Biszczanik A., Walesa K., Kukla M., Górecki J. (2021). The influence of Density on the Value of Young’s Modulus for Dry Ice. Materials.

[30] Górecki J., Malujda I., Talaska K., Tarkowski P., Kukla M. (2016). Influence of the value of limit density stress on the quality of the pellets during the agglomeration process of CO_2_. Procedia Eng..

[31] Górecki J., Wiktor L. (2023). Influence of Die Land Length on the Maximum Extrusion Force and Dry Ice Pellets Density in Ram Extrusion process. Materials.

[32] Marques F., Flores P., Claro J.C.P., Lankarani H.M. (2016). A survey and comparison of several friction force models for dynamic analysis of multibody mechanical systems. Nonlinear Dyn..

[33] Southwards S.C., Radcliffe C.J., MacCluer C.R. (1991). Robust nonlinear stick-slip friction compensation. J. Dyn. Syst. Meas. Control.

[34] Mundry R., Nunn C. (2009). Stewise Model Fitting and Statistical Inference: Turning Noise into Signal Pollution. Am. Nat..

[35] Shah M., Fazil S., Ali S., Pandey Y., Faisal S., Mehraj I. (2017). Modeling of Runoff using Curve Expert for Dachigam-Telbal Catchment of Kashmir Valley, India. Int. J. Curr. Microbiol. Appl. Sci..

[36] Middelhoff M., Cuisinier O., Masrouri F., Talandier J., Conil N. (2020). Combined impact of selected material properties and environmental conditions on the swelling pressure of compacted claystone/bentonite mixes. Appl. Clay Sci..

[37] Ku Q., Zhao J., Mallon G., Zhao S. (2023). Compaction of highly deformable coherent granular powders. Powder Technol..

[38] Greaves G., Greer A., Lakes R., Rouxel T. (2011). Poissons’s ratio and modern materials. Nat. Mater..

[39] Kaufmann E., Attree N., Bradwell T., Hagermann A. (2020). Hardness and Yield Strength of CO_2_ Ice under Martian Temperature Conditions. J. Geophys. Res. Planets.

[40] Xu F., Wang H., Wu X., Ye Z., Lie H. (2022). A Densoty-dependent Modified Doraivelu Model for the Cold Compaction of Poly (Ether Ketone Ketone Ketone) Powders. Polymers.

